# Detection of tau in Gerstmann-Sträussler-Scheinker disease (*PRNP* F198S) by [^18^F]Flortaucipir PET

**DOI:** 10.1186/s40478-018-0608-z

**Published:** 2018-10-29

**Authors:** Shannon L. Risacher, Martin R. Farlow, Daniel R. Bateman, Francine Epperson, Eileen F. Tallman, Rose Richardson, Jill R. Murrell, Frederick W. Unverzagt, Liana G. Apostolova, Jose M. Bonnin, Bernardino Ghetti, Andrew J. Saykin

**Affiliations:** 1 Department of Radiology and Imaging Sciences, Indiana University School of Medicine, 355 West 16th Street, Suite 4100, Indianapolis, IN 46202 USA; 2 Indiana Alzheimer Disease Center, Indiana University School of Medicine, Indianapolis, IN USA; 3 Department of Neurology, Indiana University School of Medicine, Indianapolis, IN USA; 4 Department of Psychiatry, Indiana University School of Medicine, Indianapolis, IN USA; 5 Department of Pathology and Laboratory Medicine, Indiana University School of Medicine, Indianapolis, IN USA; 6 Department of Medical and Molecular Genetics, Indiana University School of Medicine, Indianapolis, IN USA

**Keywords:** Positron emission tomography (PET), [^18^F]flortaucipir/AV-1451/T-807, Gerstmann-Sträussler-Scheinker disease (GSS), Tau, Prion protein (PrP), *PRNP* F198S mutation

## Abstract

This study aimed to determine the pattern of [^18^F]flortaucipir uptake in individuals affected by Gerstmann-Sträussler-Scheinker disease (GSS) associated with the *PRNP* F198S mutation. The aims were to: 1) determine the pattern of [^18^F]flortaucipir uptake in two GSS patients; 2) compare tau distribution by [^18^F]flortaucipir PET imaging among three groups: two GSS patients, two early onset Alzheimer’s disease patients (EOAD), two cognitively normal older adults (CN); 3) validate the PET imaging by comparing the pattern of [^18^F]flortaucipir uptake, in vivo, with that of tau neuropathology, *post-mortem*. Scans were processed to generate standardized uptake value ratio (SUVR) images. Regional [^18^F]flortaucipir SUVR was extracted and compared between GSS patients, EOADs, and CNs. Neuropathology and tau immunohistochemistry were carried out *post*-*mortem* on a GSS patient who died 9 months after the [^18^F]flortaucipir scan. The GSS patients were at different stages of disease progression. Patient A was mildly to moderately affected, suffering from cognitive, psychiatric, and ataxia symptoms. Patient B was moderately to severely affected, suffering from ataxia and parkinsonism accompanied by psychiatric and cognitive symptoms. The [^18^F]flortaucipir scans showed uptake in frontal, cingulate, and insular cortices, as well as in the striatum and thalamus. Uptake was greater in Patient B than in Patient A. Both GSS patients showed greater uptake in the striatum and thalamus than the EOADs and greater uptake in all evaluated regions than the CNs. Thioflavin S fluorescence and immunohistochemistry revealed that the anatomical distribution of tau pathology is consistent with that of [^18^F]flortaucipir uptake. In GSS patients, the neuroanatomical localization of pathologic tau, as detected by [^18^F]flortaucipir, suggests correlation with the psychiatric, motor, and cognitive symptoms. The topography of uptake in *PRNP* F198S GSS is strikingly different from that seen in AD. Further studies of the sensitivity, specificity, and anatomical patterns of tau PET in diseases with tau pathology are warranted.

**Electronic supplementary material:**

The online version of this article (10.1186/s40478-018-0608-z) contains supplementary material, which is available to authorized users.

## Introduction

Gerstmann-Sträussler-Scheinker disease (GSS) [9] is a rare dominantly inherited prion protein (PrP) amyloidosis. GSS patients, from a large kindred, have been extensively studied in three generations [13]; they carry a TTC to TCC DNA change at codon 198 of the prion protein gene (*PRNP)* resulting in a phenylalanine to serine substitution (F198S) in the prion protein [6, 7, 11, 12, 14, 16, 35]. Neuropathologic examinations in these patients have shown that the extracellular PrP amyloid coexists with a severe intraneuronal tau pathology, characterized by deposits of hyperphosphorylated tau and neurofibrillary tangles (NFT) in the cerebral gray matter, but not in the cerebellum [10, 11, 14]. Clinically, *PRNP* F198S mutation carriers present with cerebellar ataxia and dysarthria, with later bradykinesia and rigidity. These neurologic symptoms may be preceded by psychiatric manifestations including drug dependence, depression, and/or psychosis [7]. As the disease progresses, memory impairment and cognitive dysfunction become severe [35].

In the brain of individuals carrying the *PRNP* F198S mutation, PrP amyloid deposits occur in the form of multicentric plaques and diffuse deposits throughout the cerebral cortex, subcortical nuclei, cerebellum, and brainstem [10, 12]. Neuropathologically, the pattern of distribution of PrP amyloid differs substantially from that of the amyloid β (Aβ) peptide, which is the major component of the plaques in the dominantly inherited and sporadic forms of Alzheimer’s disease (AD). Limited neuropathologic data from non-symptomatic *PRNP* F198S carriers suggest that extracellular PrP amyloid deposits precede the development of tau pathology [11]. Deposition of tau occurs in the form of tau-immunoreactive intracytoplasmic deposits in neurons, NFT, and neuropil threads [10, 12]. By transmission electron microscopy and Western blot analysis, the neurofibrillary tangles in GSS associated with the *PRNP* F198S mutation are similar to those seen in AD [33]. Tau deposition occurs in close proximity to the PrP amyloid deposits, and thus, the pattern of tau pathology in the cerebrum mirrors that of PrP amyloid. As a consequence, both tau spread and topography in this prion disease differ substantially from those observed in AD and other neurodegenerative diseases with tau pathology.

Neuroimaging studies of patients with GSS have employed structural magnetic resonance imagin (MRI), positron emission tomography (PET), and single photon emission computerized tomography (SPECT). To date, only a few studies have evaluated neuroimaging measures in *PRNP* F198S GSS patients. Vitali et al. (2011) studied changes on fluid-attenuated inversion recovery (FLAIR) and diffusion-weighted imaging (DWI) in patients with GSS, including two who carried the *PRNP* F198S mutation and demonstrated hyperintense signal in *PRNP* F198S individuals in limbic, neocortical, and subcortical regions [36]. Kepe et al. (2010) evaluated alterations in GSS patients, including two symptomatic and two asymptomatic *PRNP* F198S GSS patients from the Indiana kindred, on 2-(1-(6-[(2-[fluorine-18]fluoroethyl)(methyl)amino]-2-naphthyl)-ethylidene)malononitrile ([^18^F]FDDNP) PET, [^18^F]fluorodeoxyglucose (FDG) PET, and structural MRI [19] images. In that report, the symptomatic *PRNP* F198S GSS patients and one asymptomatic carrier had increased [^18^F]FDDNP binding in the basal ganglia, thalamus, cerebral cortex, and cerebellum. Reduced metabolism and mild atrophy were also seen in similar regions in symptomatic *PRNP* F198S GSS patients. Recently, another study demonstrated that [^11^C]PiB, which is selective for Aβ deposition, showed no specific signal in asymptomatic and symptomatic individuals carrying the *PRNP* P102L mutation or the *PRNP* F198S mutation [4]. Currently, no ligand is available to specifically demonstrate PrP amyloid deposition by PET.

In this study, we sought to: 1) determine the pattern of [^18^F]flortaucipir uptake in *PRNP* F198S GSS patients; 2) compare the tau distribution on [^18^F]flortaucipir PET among the following three groups: *PRNP* F198S GSS affected individuals, sporadic early onset AD patients (EOAD), cognitively normal older adults (CN); and, 3) compare the pattern of [^18^F]flortaucipir uptake, in vivo*,* with that of tau neuropathology, *post-mortem*. Based on the neuropathological similarity of the tau NFT in *PRNP* F198S GSS and AD [33], we hypothesized that [^18^F]flortaucipir, a recently developed PET tracer that is specifically sensitive to tau NFTs [2, 37], would permit in vivo detection of tau deposits in *PRNP* F198S GSS patients. In the present study, we report, for the first time, data showing [^18^F]flortaucipir uptake in two symptomatic GSS individuals carrying the F198S *PRNP* mutation and compare the uptake patterns in these individuals with patterns observed in cognitively normal older adults (CN) and in patients with EOAD. The [^18^F]flortaucipir PET results are also validated by the neuropathologic demonstration of PrP amyloid and tau deposits in one of the two GSS patients, who died 9 months after the [^18^F]flortaucipir PET scan.

## Materials and methods

### Clinical assessment

All participants were evaluated in the context of annual research visits to the Indiana Alzheimer Disease Center (IADC). The clinical assessments included neurological examinations, structured informant interviews for symptoms and function, and neuropsychological assessments. Diagnoses were made by consensus panel using research criteria. Assessments were compliant with National Alzheimer’s Coordinating Center (NACC) procedures at the time of the visits including: demographics, health histories, medications, family histories, Clinical Dementia Rating (CDR), Functional Assessment Scale (FAS), Geriatric Depression Scale (GDS), and Neuropsychiatric Interview Questionnaire (NPI-Q). At the time of the [^18^F]flortaucipir PET scans, cognitive testing with the UDS3 measures included: Montreal Cognitive Assessment (MoCA), Craft Stories immediate and delayed recall, Benson Complex Figure copy and delayed recall, the Multilingual Naming Test (MINT), Animal fluency, Vegetable fluency, Phonemic fluency (letters F and L), Trail Making Test Parts A and B (TMT-A and TMT-B), and Number Span forward and backward. The Rey Auditory Verbal Learning Test (RAVLT) and Digit Symbol Substitution Test were also given to all participants. Written informed consent was obtained from all participants in accordance with the Declaration of Helsinki and the Belmont Report. All procedures were approved by the Indiana University School of Medicine Institutional Review Board.

### Genetics

DNA was extracted from fresh blood samples of patients A and B and the open-reading frame of the Prion Protein gene (*PRNP*) was analyzed by direct sequencing. The same procedure was used to study DNA extracted from the brain tissue of patient B and several previously deceased family members [16].

### Structural MRI

A T1-weighted magnetization-prepared rapid gradient-echo (MPRAGE) structural MRI sequence was acquired at the time of the [^18^F]flortaucipir PET scan on a 3 Tesla Siemens Prisma scanner for both patients. Automatic parcellation with Freesurfer version 5.1 (https://surfer.nmr.mgh.harvard.edu/fswiki/FreeSurferWiki) was completed to create subject-specific regions of interest (ROIs) to use for extraction of mean [^18^F]flortaucipir standardized uptake value ratio (SUVR; see [^18^F]Flortaucipir section) from target regions. MPRAGE scans were also segmented in Statistical Parametric Mapping 8 (SPM8) to generate subject-specific spatial normalization parameters for use in PET scan processing.

### [^18^F]Flortaucipir PET

Both patients were studied with [^18^F]flortaucipir PET within two months of the nearest clinical visit (mean age in the sixth decade). They were injected intravenously with approximately 10 mCi of [^18^F]flortaucipir and after a 75-min uptake period were scanned for 30 min on a Siemens mCT (six 5-min frames). Scans were reconstructed according to the Alzheimer’s Disease Neuroimaging Initiative (ADNI)-2 protocol (http://adni.loni.usc.edu/wp-content/uploads/2015/02/01_DOD-ADNI_Tau-Addendum-Protocol_23Oct2014.pdf). Standard processing, including spatial alignment for motion and normalization to Montreal Neurologic Institute (MNI) space using parameters from the MRI segmentation, was completed in SPM8. Mean static images from 80 to 100 min post-injection were generated by averaging the appropriate frames and smoothed with an 8 mm full-width half maximum (FWHM) Gaussian kernel. Finally, [^18^F]flortaucipir SUVR images were generated by intensity normalizing by mean cerebellar crus uptake. The [^18^F]flortaucipir PET scans were qualitatively visualized using MRIcron (http://www.mccauslandcenter.sc.edu/mricro/mricron/). Mean [^18^F]flortaucipir SUVR values were extracted from subject-specific ROIs, including the caudate nucleus, putamen, pallidum, thalamus, insula, anterior cingulate gyrus, posterior cingulate gyrus, overall lobar regions (frontal, parietal, temporal, and occipital), the overall cingulate cortex, the sensory-motor cortices, and the global cortex.

[^18^F]Flortaucipir PET scans from two CN (mean age of approximately 67.5 years) and two Aβ-positive EOAD patients (mean age of approximately 61 years; mean age of onset of approximately 59 years) were used as comparisons. The patients were selected to match the *PRNP* F198S GSS patients by sex and, as closely as possible, by age and global cognition on the MoCA. All scans were processed as described above. Scans were visualized using MRIcron and mean SUVR was extracted from Freesurfer-generated subject-specific ROIs for the regions described above. For display purposes (Fig. 4), mean SUVR values were calculated for the target ROIs in both CN individuals and both EOAD patients as comparisons to the *PRNP* F198S GSS patients.

### Neuropathology

Patient B expired 9 months after the [^18^F]flortaucipir PET scan. The brain, harvested at Indiana University School of Medicine, was hemisected along the mid-sagittal plane. The left hemibrain was fixed in formalin. Following fixation in a 10% formalin solution, the left cerebral and cerebellar hemispheres, as well as the left half of the brainstem, were sliced and tissue samples were selected. In order to compare neuropathology with tau PET imaging, six hemispheric coronal slabs were selected that included areas of the frontal, insular, temporal, parietal, and occipital lobes. These were submitted in their entirety for histology and immunohistochemistry. In addition, blocks of the following CNS areas were also submitted: superior frontal gyrus, middle frontal gyrus, anterior cingulate gyrus, superior temporal gyrus, middle temporal gyrus, hippocampus at two levels, entorhinal cortex, precentral cortex, postcentral cortex, inferior parietal lobule, posterior cingulate gyrus and precuneus, calcarine cortex, caudate nucleus, putamen, globus pallidus, amygdala, claustrum, thalamus, subthalamic nucleus, cerebellar vermis, cerebellar cortex and dentate nucleus, midbrain, pons, medulla, and spinal cord at cervical, thoracic, lumbar, and sacral levels. The areas submited were representative of the following Brodmann Areas: 1, 2, 3, 4, 5, 6, 7, 8, 9, 11, 12, 17, 18, 19, 20, 21, 22, 23, 24, 27, 28, 31, 32, 36, 37, 38, 44, 47. The right hemibrain was sliced, frozen, and stored at − 70 °C for structural, biochemical and molecular genetic studies of PrP and tau and for the analysis of the seeding properties of PrP and tau in *PRNP* F198S GSS.

Brain tissue samples from the left hemibrain were dehydrated in graded alcohols, cleared in xylene, and embedded in paraffin. Eight-micrometer-thick sections from multiple brain areas were stained using the histological and immunohistochemical methods described below. Hematoxylin and eosin (H&E) and Luxol fast blue-hematoxylin & eosin (LFB-H&E) were used to survey gray and white matters for neuronal loss, gliosis, vascular pathology, and other possible pathologic lesions. The Thioflavin S method was used to visualize amyloid deposits and neurofibrillary tangles. Prussian blue stain enhanced by DAB (Prussian Blue-DAB) visualized the ferric iron deposits in the tissue. Neurodegenerative pathology was further analyzed using antibodies raised against PrP (3F4, 1:800, Dr. Richard Kacsak, Staten-Island, New York, USA), tau (AT8, 1:300, Thermo Fisher Scientific, Waltham, MA, USA; PHF-1, 1:10, gift of Dr. P. Davies); 3-repeat tau (3R, 1:3000, Millipore, Billerica Massachusetts, USA), 4-repeat tau (4R, 1:100, Millipore, Billerica Massachusetts, USA), anti-phospho-TDP-43 (1:1000, Cosmo Biologicals, Carlsbad, CA, USA), α-synuclein (ASy119–137, 1:300, Dr. P. Piccardo, Dr. B. Ghetti) and amyloid β (Aβ 21F12, 1:1000, Janssen Research & Development, South San Francisco, CA, USA). AT8 recognizes tau phosphorylated at serine 202 and threonine 205, while PHF-1 recognizes tau phosphorylated at serine 396 and serine 404. The signal from polyclonal or monoclonal antibodies was visualized using avidin-biotin, with goat anti-rabbit immunoglobulin or goat anti-mouse as the secondary antibody as required, followed by horseradish peroxidase-conjugated streptavidin and the chromogen diaminobenzidine. Immunohistochemical sections were counterstained with hematoxylin.

## Results

### Case histories

#### Patient A

Patient A had a family history of GSS and completed 15 IADC clinical yearly assessments before the tau PET scan. The subject was right-handed, had a few years of college education, and was first examined neurologically in their fourth decade. At the first twelve annual visits, clinical, neurologic, and neuropsychological assessments were within normal limits in all respects. During this time period, Patient A was found to have the *PRNP* F198S mutation. In the sixth decade, Patient A developed mild depression and the patient’s informant reported the onset of a gradually progressive memory impairment accompanied by a change in personality, characterized by sadness and withdrawal developing over an 18-month period. The NPI-Q also indicated mild apathy and irritability. Otherwise, the neurological and neuropsychological exams were unremarkable and the patient was determined to be cognitively normal. Approximately 1 year later, the patient’s informant reported mild worsening of the psychiatric symptoms, including mild depression, irritability, and changes in motor behaviors. Neurologically and cognitively, however, the patient was considered normal except for a mild decline in psychomotor speed that remained within the normal range for the patient’s age. The neurological examination on the next visit, approximately 1 year later,was again unremarkable but the supplemental CDR for the behavioral, comportment, and personality domains now indicated mild impairment (a 0.5 rating). NPI-Q revealed progression of symptoms, including mild changes in motor behaviors and mild ataxia, moderate depressive and anxiety symptoms, and altered nighttime behaviors and appetite. The neuropsychological battery indicated mild decline in psychomotor speed and complex sequencing, but these were still within the normal range. Normal cognition was again the consensus diagnosis.

The [^18^F]flortaucipir PET and the structural MRI scans were completed at the sixteenth clinical assessment. The informant reported a continued decline in memory, progressive changes in personality, as well as slowly progressive decline in language. At the neurological examination, gait abnormalities, slowness, and falls were observed. Results of the neuropsychological examination are shown in Table 1. The global CDR was 0.5, indicating mild global impairment, with mild impairment (a 0.5 rating) in the memory domain and mild-to-moderate impairment (a 1.0 rating) in the judgement and problem-solving domain. The FAS indicated difficulty understanding books and TV shows, difficulty remembering appointments and medications, and need for assistance with finances. The NPI-Q again showed mild depressive symptoms, changes in motor behavior, and changes in appetite. The neuropsychological assessment revealed a decline in global cognitive status, moderate impairment in complex sequential tracking and psychomotor speed, moderate impairment in manual motor skills, and mild impairment in new learning and memory. Self-reported mood was within normal limits on the GDS. The consensus diagnosis at this visit was mild cognitive impairment due to GSS. One year following the tau PET scan (the 17th clinical assessment), Patient A showed progression of neurologic, psychiatric, and cognitive symptoms; the informant reported continued gradually progressive decline in memory, language, judgment, reasoning, and attention. The consensus diagnosis at that time was mild dementia due to GSS.

**Table 1:** Neuropsychological performance of participants at the time of the [^18^F]flortaucipir scan

	GSS Participants		Early-Onset Alzheimer’s		Cognitive Normals	
	Patient A	Patient B	Patient #1	Patient #2	#1	#2
MoCA	22	18	18	17	29	26
CDR Global	0.5	1	0.5	0.5	0	0
CDR Sum of Boxes	1.5	7	3	3.5	0	0
Digit Span Forward	10	5	10	8	9	9
Digit Span Backward	8	5	10	6	12	10
Trail Making Test A	49	76	53	35	23	20
Trail Making Test B	129	300	209	159	61	38
WAIS Digit Symbol	36	20	40	27	68	82
Animal Fluency	15	8	13	21	24	23
Vegetable Fluency	9	11	11	3	18	13
Letter Fluency	25	11	36	26	36	25
MINT	31	28	27	30	32	24
RAVLT Immediate	41	22	27	22	56	51
RAVLT Delayed	7	3	0	0	11	8
Craft Stories Immediate	9	5	6	5	18	15
Craft Stories Delayed	10	4	2	0	17	16
Benson Figure Copy	16	10	14	17	15	15
Benson Figure Recall	10	8	2	0	12	13
GDS	4	4	2	2	1	0
FAS	7	13	14	17	1	0
NPI-Q	3	10	7	9	1	2
Finger Tapping – Dom	39	24	48	58	39	42
Finger Tapping – ND	33	25	36	52	43	40

*CDR* Clinical Dementia Rating scale, *Dom* dominant hand, *FAS* Functional Assessment Scale, *GDS* Geriatric Depression Scale, *GSS* Gerstmann-Sträussler-Scheinker disease, *MINT* Multi-lingual Naming Test, *MoCA* Montreal Cognitive Assessment, *ND* non-dominant hand, *NPI*-*Q*Neuropsychiatric Inventory Questionnaire, *RAVLT* Rey Auditory Verbal Learning Test, *WAIS* Wechsler Adult Intelligence Scale

#### Patient B

Patient B had a family history of GSS, was first assessed at the IADC in the sixth decade, and was found to have the *PRNP* F198S mutation. At time of the examination, the informant reported a one-year history of gradually progressive memory difficulties and a language disorder, as well as a six-month history of difficulties with judgment and reasoning, accompanied by changes in personality characterized by irritability, withdrawal, sadness, socially inappropriate behavior, and agitation. On neurological examination, there was slight tremor, bradykinesia, and ataxic gait. The global CDR was 0.5, indicating mild global impairment, with mild impairment (a 0.5 rating) in the memory, orientation, judgement and problem-solving, community affairs, and home and hobbies domains, and moderate impairment (a 1.0 rating) in the behavior, comportment, and personality domains of the supplemental CDR. The NPI-Q indicated the presence of agitation, depression, anxiety, disinhibition, irritability, nighttime behaviors, and problems with appetite, with severity scores predominantly in the mild range. The FAS indicated mild impairment in daily functioning, including shopping, playing games, using and turning off the stove, meal preparation, keeping track of current events, remembering dates, and traveling out of the neighborhood. The neuropsychological battery revealed mild impairments in efficiency of new learning, verbal fluency, response inhibition, complex sequential tracking, and manual motor speed. The Mini-Mental State Examination score was 26/30. The GDS was within normal limits. The consensus diagnosis was mild dementia due to GSS.

At the follow-up IADC assessment approximately 1 year later, the informant reported continued loss of memory, language, judgment, and reasoning, as well as continued deterioration in personality, including the appearance of delusions. On neurological examination, there was evidence of parkinsonism and slowness, with frequent falls and gait disturbance. Results of the neuropsychological examination are shown in Table 1. The global CDR had worsened to 1.0, indicating mild impairment, with very mild impairment (a 0.5 rating) in memory and orientation domains, mild to moderate impairment (a 1.0 rating) in judgement and problem-solving, community affairs, and personal care domains, and severe impairment (a 3.0 rating) in the home and hobbies domain. The NPI-Q now included delusions in addition to the problems noted on the previous visit, with most symptoms rated as moderate in severity. The FAS total score had worsened to 13, with need for assistance in shopping, simple meal preparation and cooking, and traveling. The second neuropsychological examination revealed mild to moderate declines in new learning, memory, executive cognitive function, psychomotor speed, and manual motor skills. The MoCA score was 18/30 (equivalent to a MMSE score of 24/30 [34]) and GDS was within normal limits. The consensus diagnosis was dementia due to GSS. The [^18^F]flortaucipir PET and the structural MRI scans were completed at this visit.

Patient B died while at hospice care 9 months after the completion of the [^18^F]flortaucipir PET scan from progression of the GSS dementia.

### Early-onset AD patient #1

EOAD patient #1 was found to be amyloid positive on a [^18^F]florbetapir PET scan and showed medial temporal and global atrophy on the structural MRI. At the time of the [^18^F]flortaucipir PET scan, EOAD patient #1 was aged in the seventh decade and had a global CDR of 0.5, indicating mild impairment, with mild impairment (a 0.5 rating) in the orientation and judgment and problem-solving domains and mild to moderate impairment (a 1.0 rating) in the memory and home and hobbies domains, but normal functioning (a 0.0 rating) in the behavioral, comportment, and personality domains of the supplemental CDR. The informant reported a four-year history of gradually progressive memory problems, a 1 year history of gradually progressive problems with judgment and reasoning, and a 3–4 month history of gradually progressive problems with attention and concentration. There were no neurological abnormalities. Results of the neuropsychological examination are shown in Table 1. The NPI-Q revealed depression and anxiety at a moderate level and agitation, irritability, and abnormal nighttime behaviors at a mild level of severity. The FAS total score was 14, indicating mild difficulties with finances, shopping, meal preparation, remembering appointments, pastimes, current events, and travel outside of the neighborhood. The neuropsychological battery revealed moderate impairments in global cognitive status, complex sequential tracking, and psychomotor speed, and severe impairment in new learning and memory. The consensus diagnosis was dementia due to AD [25].

### Early-onset AD patient #2

EOAD patient #2 was found to be amyloid positive on a [^18^F]florbetapir PET scan and showed medial temporal and global atrophy on the structural MRI. At the time of the [^18^F]flortaucipir PET scan, EOAD patient #2 was aged in the sixth decade and had a global CDR of 0.5, indicating mild impairment, with mild impairment (a 0.5 rating) in the judgment and problem-solving domain and mild to moderate impairment (a 1.0 rating) in the memory, orientation, and home and hobbies domains, and mild impairment (a 0.5 rating) the behavioral, comportment, and personality domains of the supplemental CDR. The informant reported a three-year history of gradually progressive memory loss and a two-year history of gradually progressive difficulty with judgment, reasoning, and attention. There were no abnormal neurological manifestations. Results of the neuropsychological examination are shown in Table 1. The NPI-Q reflected mild problems with delusions, depression, indifference, disinhibition, irritability, abnormal nighttime behaviors, and problems with eating. The FAS total score was 17, indicating a substantial impairment and dependency in many activities of daily living. The neuropsychological battery revealed moderate impairment in global cognitive status and severe impairment in new learning and memory. There were moderate impairments in complex sequential tracking. The consensus diagnosis was mild dementia due to AD.

### Cognitively Normal individuals

At the time of their [^18^F]flortaucipir PET scans, both CN individuals had global CDRs and Sum of Boxes scores of 0.0, with no informant-rated impairments or depression. On examination, there were no neurological or neuropsychological abnormalities and consensus diagnoses were normal cognition. Results of the neuropsychological examination are shown in Table 1.

### Genetics

There was a single nucleotide (T to C) substitution in codon 198 of one allele of Patient A’s and Patient B’s *PRNP* genes. This change results in a serine for phenylalanine amino acid change (F198S). For patient A, the first base of *PRNP* codon 129 was heterozygous guanine/adenine (G/A), coding for valine/methionine (GTG/ATG). For patient B, the first base of *PRNP* codon 129 was homozygous guanine (G), coding for valine (GTG).

### [^18^F]Flortaucipir PET scans in *PRNP* F198S GSS patients

**Fig. 1:**
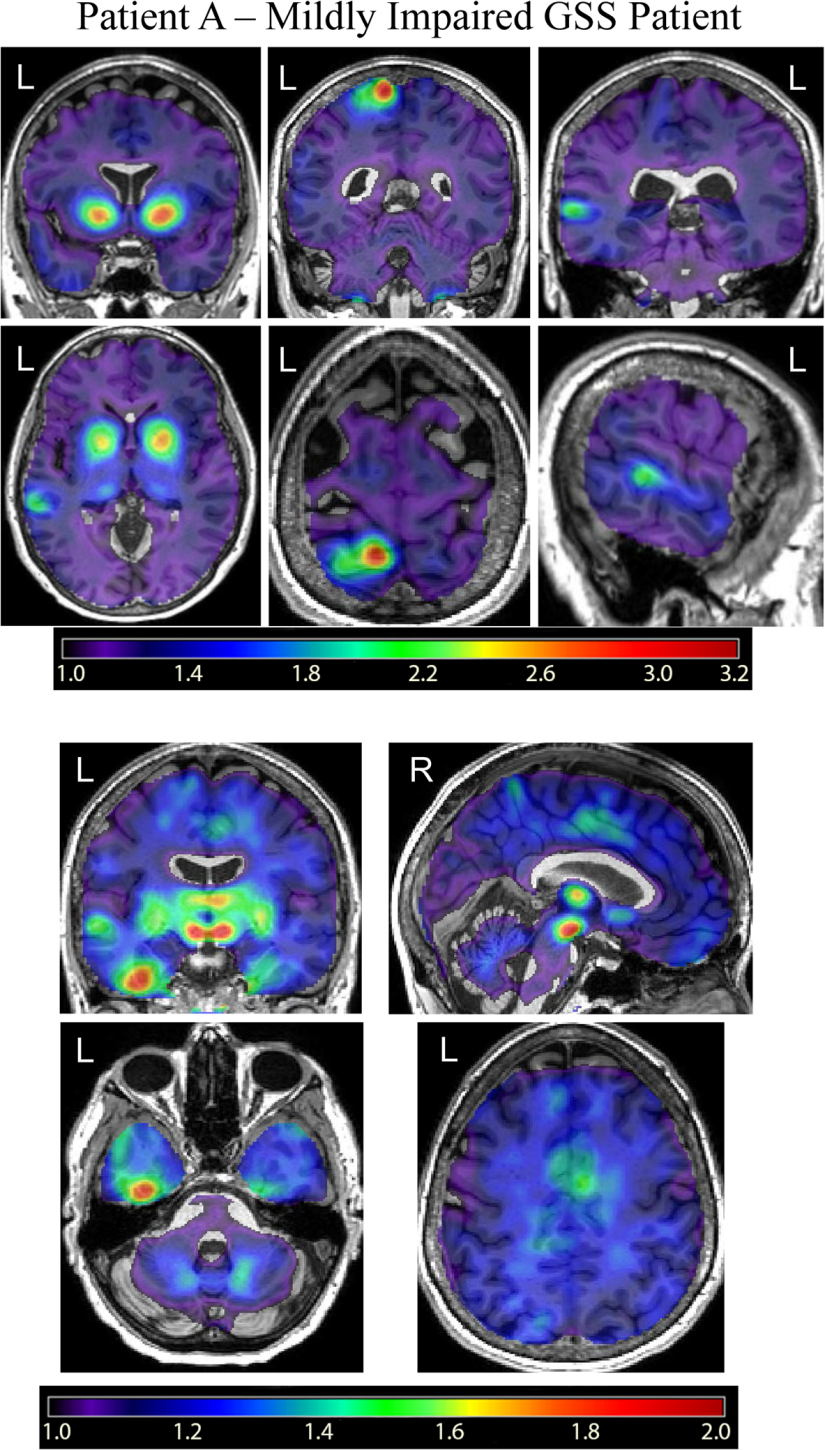
[^18^F]Flortaucipir PET in Patient A – Mildly to Moderately Impaired *PRNP* F198S GSS Patient. [^18^F]Flortaucipir scans from a mildly to moderately impaired GSS *PRNP* F198S mutation carrier (Patient **a**) show specific binding of the tracer in the basal ganglia, thalamus, insular cortex, postcentral gyrus, and inferior temporal lobe. At a lower threshold (lower panels), additional binding is seen in the medial temporal lobe and cingulate gyrus.

**Fig. 2:**
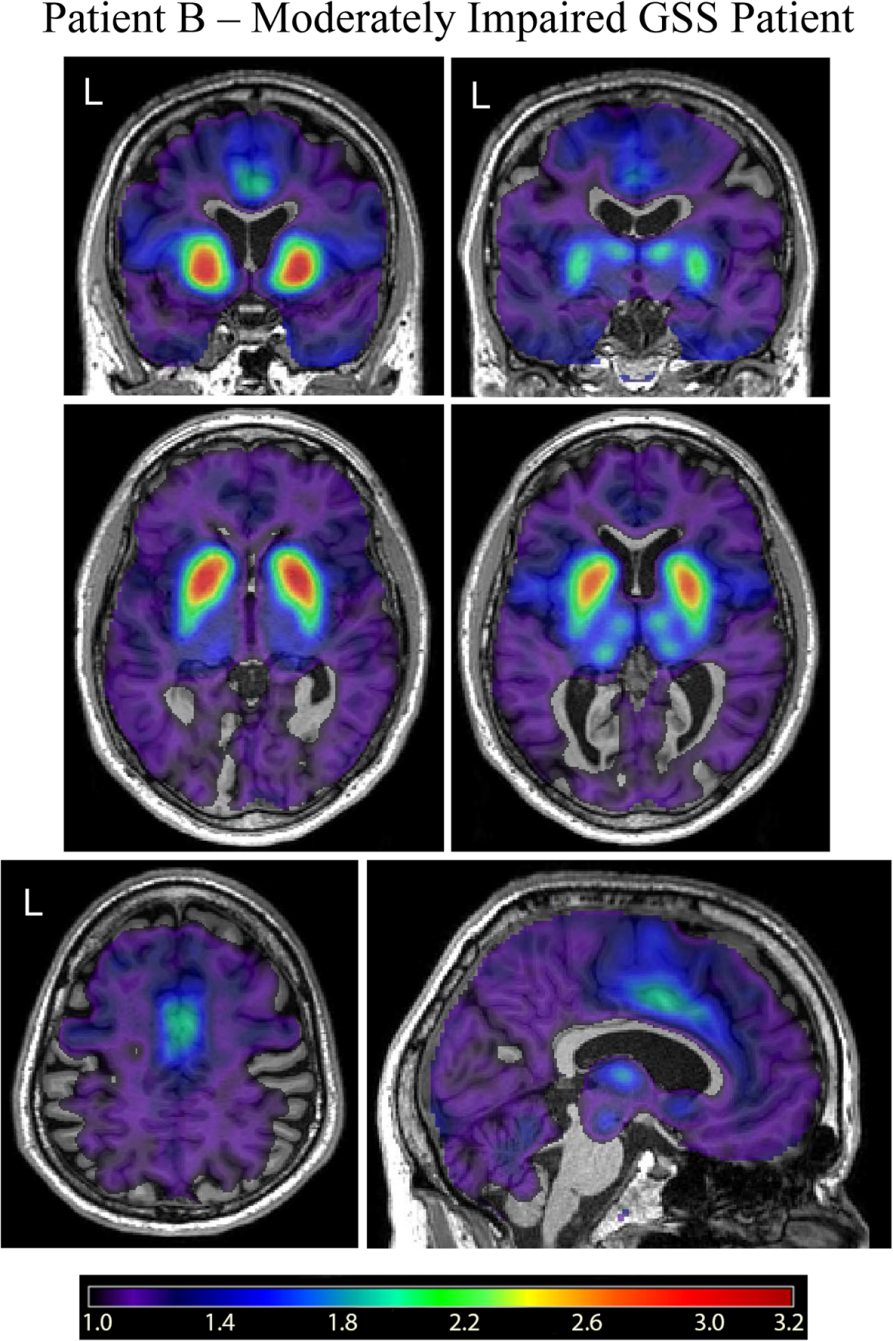
[^18^F]Flortaucipir PET in Patient B – Moderately to Severely Impaired *PRNP* F198S GSS Patient. [^18^F]Flortaucipir scans from a moderately to severely impaired GSS *PRNP* F198S mutation carrier (Patient **b**) show specific binding of the tracer in the basal ganglia, thalamus, cingulate gyrus, frontal lobe, and insula/claustrum

The [^11^C]PiB PET scans showed no specific uptake in the Patients A and B, as previously reported ([4]; Additional file 1: Figure S1). In contrast, the [^18^F]flortaucipir PET scans showed significant uptake in multiple regions in both GSS patients (Figs. 1, 2, and 4). As compared with Patient A (Fig. 1), Patient B (Fig. 2) showed a more severe clinical presentation and a wider spread of [^18^F]flortaucipir uptake. GSS Patient A also showed evidence of tracer uptake in regions (Fig. 1) that were not seen in Patient B, including the primary sensory cortex and the left temporal cortex. This [^18^F]flortaucipir uptake was determined to be within cortical tissue and did not correspond to any lesion observable on MRI. On visual inspection, both individuals showed uptake in the basal ganglia, cingulate gyrus, insular cortex, and thalamus.

### Comparison of [^18^F]Flortaucipir between GSS patients, Alzheimer’s patients, and cognitively normal individuals

**Fig. 3:**
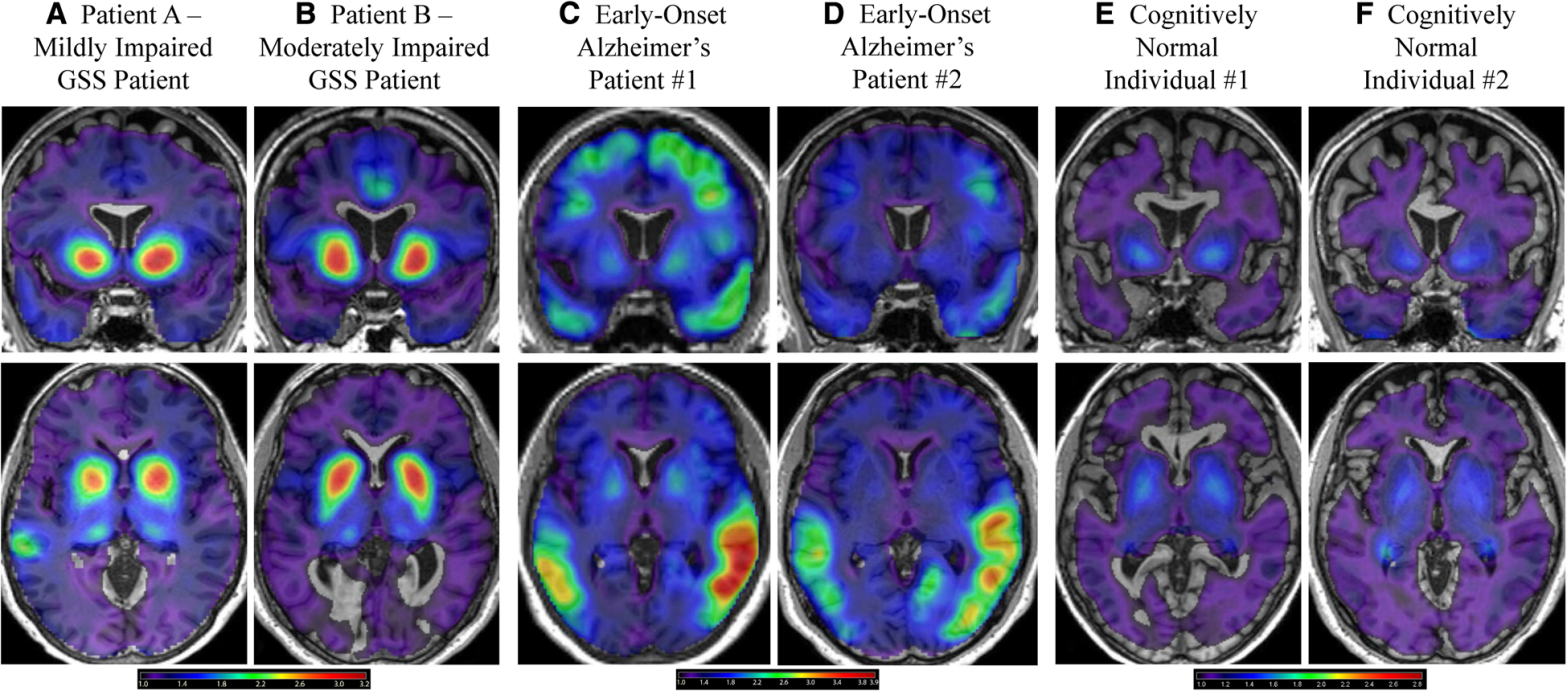
Qualitative Comparison of [^18^F]Flortaucipir PET in *PRNP* F198S GSS Patients Relative to Early-Onset Alzheimer’s Patients and Cognitively Normal Older Adults. Comparisons of selected [^18^F]flortaucipir sections of the two GSS patients with two early-onset Alzheimer’s disease (EOAD) patients and two cognitively normal older adults (CN) are shown. Higher [^18^F]flortaucipir uptake is observed in the basal ganglia of the *PRNP* F198S GSS patients (**a, b**) relative to the EOAD patients (**c, d**) and CN individuals (**e, f**). In the *PRNP* F198S GSS patients, there is also increased uptake in the thalamus relative to the EOAD patients and CN individuals. Greater uptake in the cingulate gyrus bilaterally is observed in *PRNP* F198S GSS Patient B (**b**) relative to EOAD Patient #2 (**d**) and both CN individuals (**e, f**). There is slightly increased uptake in the anterior insula bilaterally for *PRNP* F198S GSS Patient B (**b**) relative to EOAD Patient #2 (**d**) and both CN individuals (**e,f**)

Selected [^18^F]flortaucipir sections of the two GSS patients, two EOAD patients, and two CN older adults are shown in Fig. 3. Qualitatively, dramatically higher [^18^F]flortaucipir uptake was observed in the basal ganglia of Patient A (Fig. 3a) and Patient B (Fig. 3b) relatively to the EOAD (Fig. 3c, d) and CN (Fig. 3e, f) individuals. Less dramatic, but still notable, increased uptake in the thalamus was observed in both GSS patients relative to the EOAD and CN individuals. Further, greater uptake in the cingulate gyrus, bilaterally, and in the insula, anteriorly, was observed in GSS Patient B relatively to both CN individuals. Alternatively, the EOAD patients showed much higher temporal, parietal, and frontal lobes [^18^F]flortaucipir uptake than the GSS patients and the CN individuals.

**Fig. 4:**
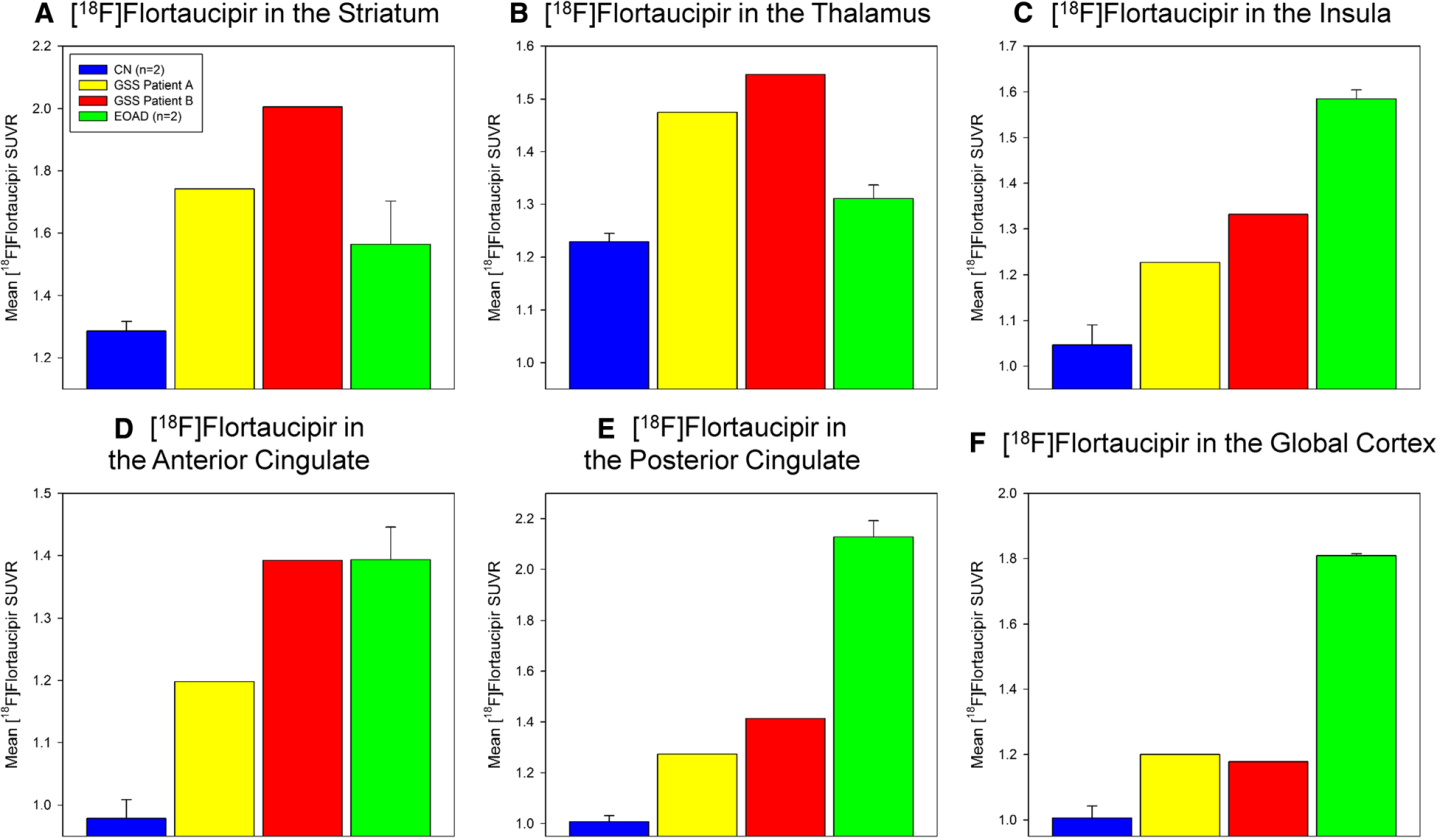
Regional Quantitative Comparisons of [^18^F]Flortaucipir SUVR in *PRNP* F198S GSS Patients Relative to Early-Onset Alzheimer’s Patients and Cognitively Normal Older Adults. Both *PRNP* F198S GSS patients showed increased mean [^18^F]flortaucipir SUVR in subcortical areas, including the striatum (**a**) and thalamus (**b**) relative to early-onset Alzheimer’s disease (EOAD) patients and cognitively normal (CN) individuals. Cortical areas such as the insula (**c**), anterior cingulate gyrus (**d**), posterior cingulate gyrus (**e**), and cortex global value (**h**) also showed increased mean [^18^F]flortaucipir SUVR in the *PRNP* F198S GSS participants relative to the CN individuals, but not the EOAD patients

Quantitative analysis confirmed much of the qualitative observation of increased [^18^F]flortaucipir uptake in GSS patients. Both GSS patients showed increased mean [^18^F]flortaucipir SUVR in subcortical areas, including the striatum and thalamus (Fig. 4a & b**;** Additional file 2: Table S1). These areas were increased relative to both the EOAD and CN individuals. Cortical areas such as insula, anterior cingulate, posterior cingulate, and, globally, in the cortical grey matter (Fig. 4c-f**;** Additional file 2: Table S1) also showed increased mean [^18^F]flortaucipir SUVR in the GSS patients relative to the CN individuals. However, the EOAD patients showed considerably greater mean [^18^F]flortaucipir SUVR in the majority of cortical regions than either the CN individuals or GSS patients.

### Neuropathology

The fresh brain of Patient B weighed 1452.5 g. There was no appreciable atrophy of the cerebral hemispheres and no focal lesions or atheromatous changes were noted on external examination. The left cerebral hemisphere was cut into twenty-seven coronal slices. The cerebellar cortex and the dentate nucleus appeared atrophic.The substantia nigra and the locus coeruleus were moderately to severely depigmented. The inferior olivary nuclei were atrophic.

**Fig. 5:**
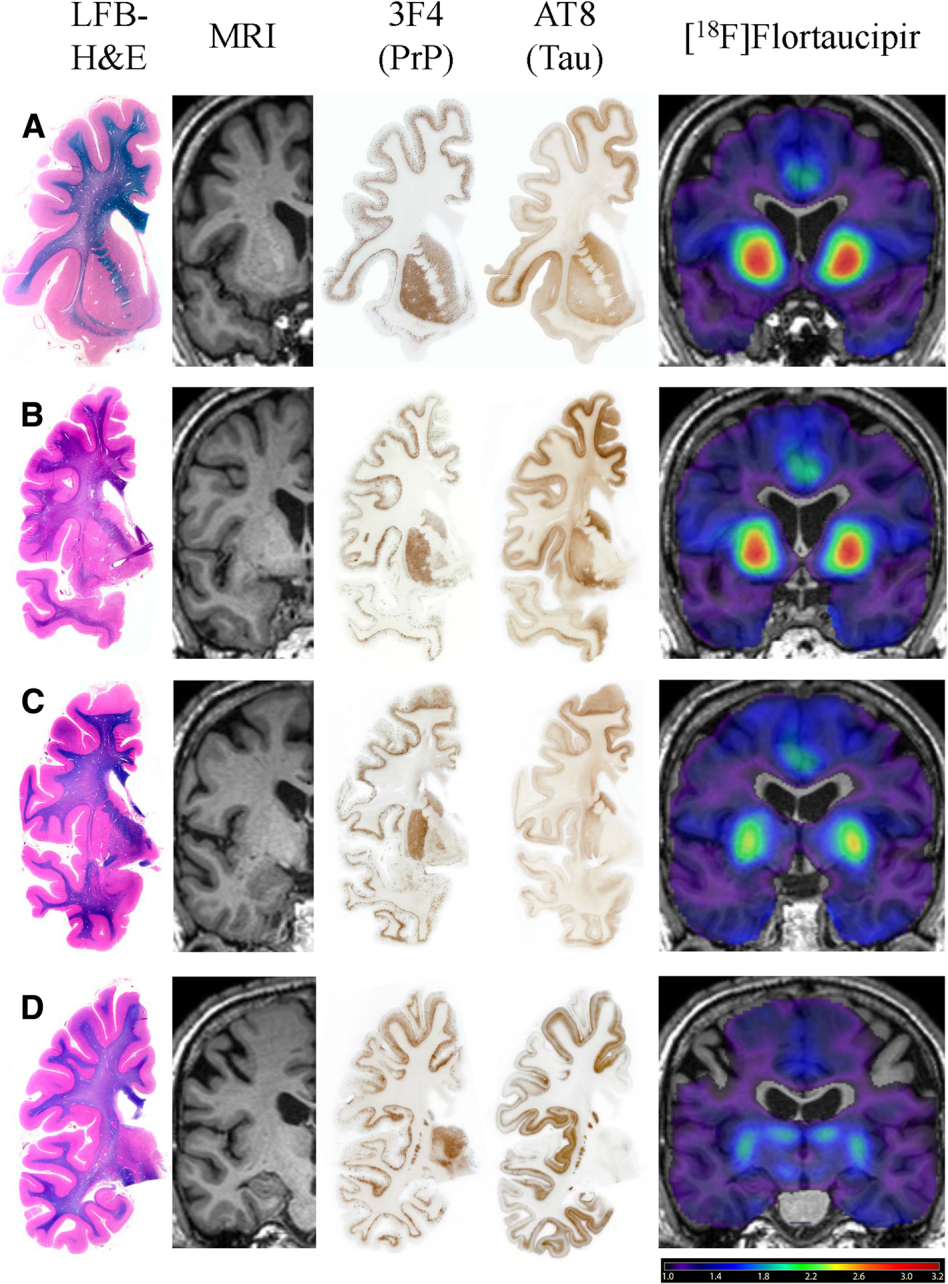
Neuropathologic Analysis of the Moderately to Severely Impaired GSS Patient B. Good correspondence between the [^18^F]flortaucipir SUVR (5th column) and AT8 immunolabeling of tau (4th column) was observed across multiple regions, including the basal ganglia and cingulate gyrus (**a-c**), as well as the frontal (**a-d)** and insular cortices (**b**,**c,d**). These areas also feature considerable structural atrophy on MRI (1st column) and LFB-H&E stain (2nd column), as well as PrP amyloid deposition on 3F4 (3rd column). The only region of non-correspondence occurred in the thalamus (**d**), where [^18^F]flortaucipir PET (5th column) showed increased uptake but no AT8 immunolabeling of tau (4th column) was observed; PrP deposition was observed in the thalamus (3rd column). LFB-H&E = luxol fast blue with hematoxylin & eosin; MRI = magnetic resonance imaging; PrP = prion protein

Serial coronal sections, stained with LFB-H&E, revealed mild and global cerebral atrophy, neuronal loss and gliosis in the gray matter, as well as a diffuse loss of myelin stain in the deep white matter of the frontal, temporal, and parietal lobes (Fig. 5, 1^st^ **column**).

**Fig. 6:**
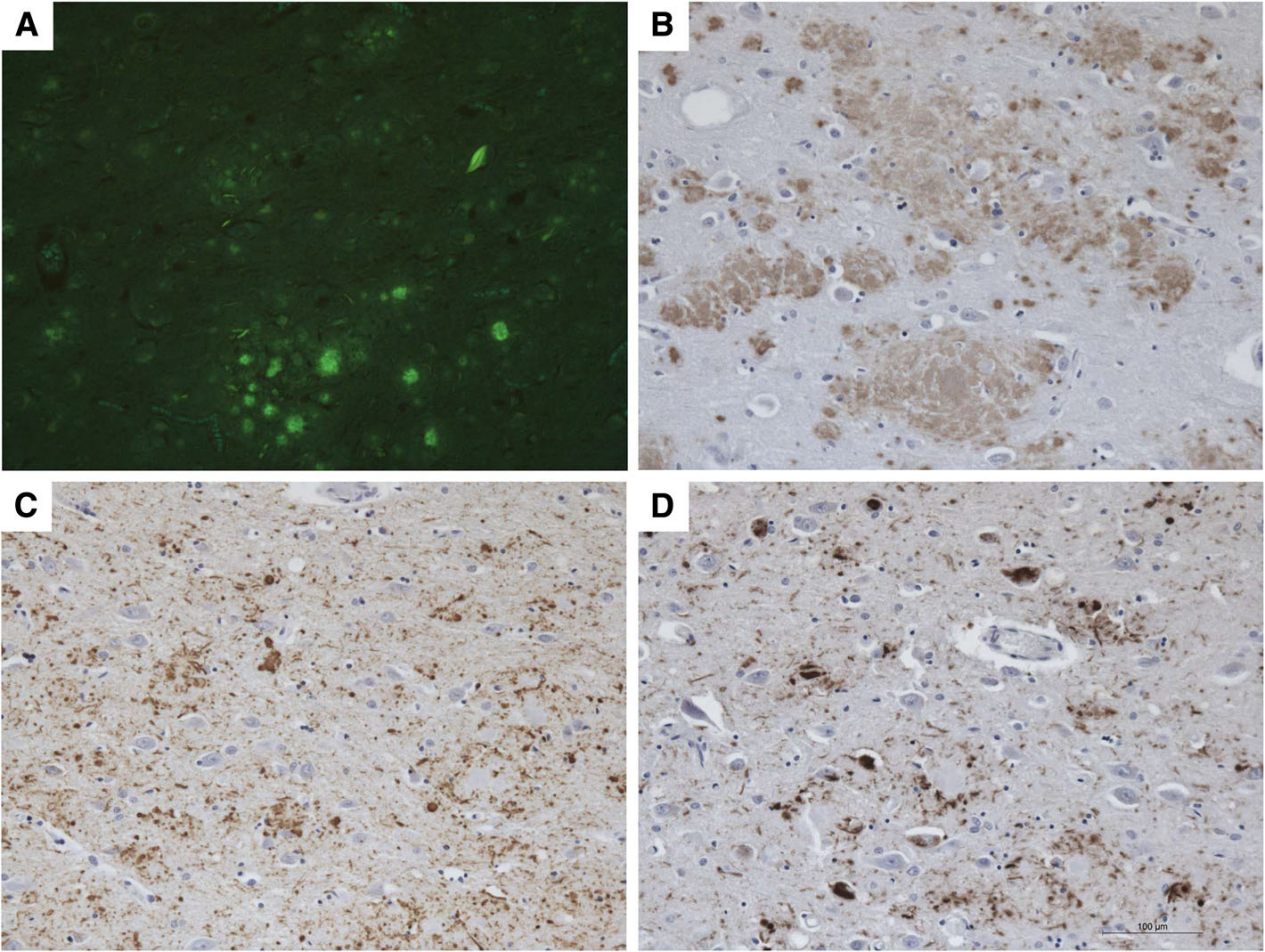
PrP and Tau in the Frontal Lobe of the Moderately to Severely Impaired GSS Patient B*.* PrP amyloid plaques and neurofibrillary tangles (NFTs) were observed using Thioflavin S in the frontal lobe (**a**). Significant immunolabeling of PrP amyloid (**b**; 3F4) and tau (**c**; AT8; **d**; PHF-1) was also observed

In Thioflavin S-stained sections of cerebrum and cerebellum, diffuse plaques and plaques with multicentric cores were numerous (Fig. 6a). The diffuse deposits were not fluorescent, but appeared brighter than the surrounding neuropil; the cores of the plaque were fluorescent and, therefore, have the tinctorial properties of amyloid. In the Thioflavin S preparations of the cerebrum, but not in preparations of the cerebellum, NFTs were seen within neuronal perikarya and in neurites that surround the amyloid cores.

Serial sections, adjacent to those stained with Thioflavin S, were immunostained for PrP and revealed that both diffuse plaques and plaques with amyloid core were decorated by PrP antibodies (Fig. 6b). By PrP immunohistochemistry, labeling of the gray matter structures of the cerebral hemisphere and of the cerebellum was observed. PrP immunopositive diffuse and multicentric cored plaques were extensively distributed in the neuropil. No intracellular PrP inclusions were present. Within the gray matter of the cerebral hemisphere, the most severe PrP immunolabeling was seen in the superior, middle, and inferior frontal gyri, the cingulate gyrus, the pre- and post-central gyri, the superior, middle and inferior temporal gyri, the fusiform gyrus, the entorhinal cortex, the parahippocampal gyrus, as well as the upper portion of the insular cortex, the caudate nucleus, putamen, and thalamus (Fig. 5, 3^rd^ **column;** Additional file 1: Figure S1).

**Fig. 7:**
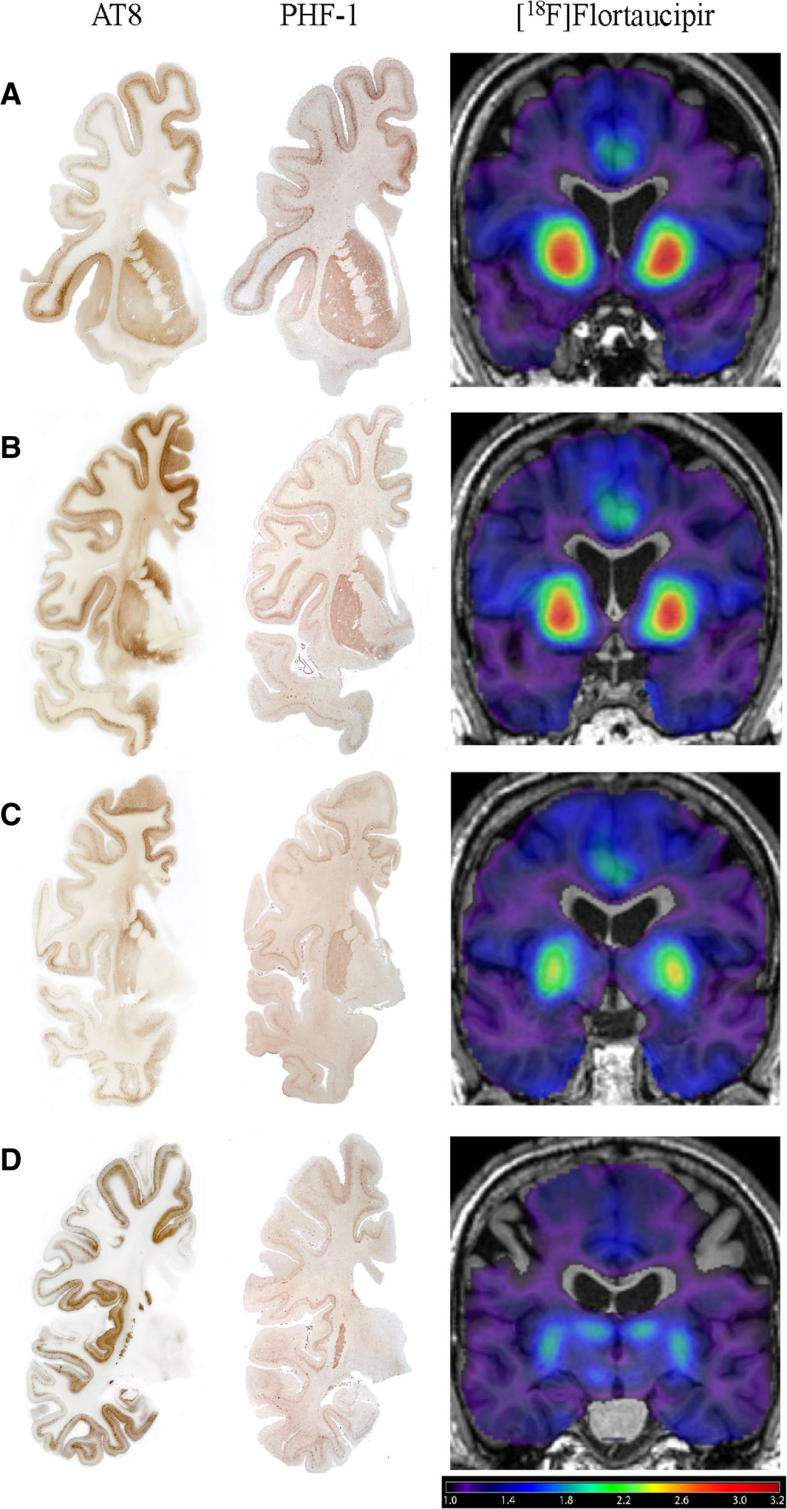
Comparison of AT8 and PHF-1 Staining to [^18^F]Flortaucipir in the Moderately to Severely Impaired GSS Patient B. Good correspondence between the [^18^F]flortaucipir SUVR (3rd column) and both the AT8 (1st column) and PHF-1 (2nd column) immunolabeling of tau and neurofibrillary tangles, respectively, was observed across the basal ganglia and cingulate gyrus (**a-c**), as well as the frontal (**a-d)** and insular cortices (**b**,**c,d**). However, while the AT8 immunolabeling was more widespread throughout the cortical and subcortical regions, the PHF-1 immunolabeling was more restricted to areas that corresponded to increased [^18^F]flortaucipir signal, with the exception of the thalamus

Serial sections, adjacent to those stained with Thioflavin S and to those immunolabeled for PrP were immunostained for tau. Decorated by the monoclonal antibodies AT8 and PHF-1 were not just the NFTs, but also multiple structures, including cytoplasm of neuronal perikarya, dentritic processes, neuropil threads, and neurites surrounding cores of plaques (Fig. 6c & d). In fact, using AT8 and PHF-1, the pattern of hyperphosphorylated tau immunohistochemical labeling mirrored that of PrP throughout the cerebral cortex and the subcortical nuclei except in the thalamus, where PrP immunoreactivity was much stronger than tau immunoreactivity (Fig. 5, 4^th^ **column;** Fig. 7**;** Additional file 1: Figure S1). Tau deposits were most numerous in the superior, middle and inferior frontal gyri, inferior temporal, fusiform, and cingulate gyri, as well as in the insular, parahippocampal, and entorhinal cortices, caudate nucleus, and putamen (Fig. 5, 4^th^ **column;** Fig. 7). Patient B showed a large number of NFT (Fig. 6d**,** Fig. 7). Tau deposits were not present in the cerebellar cortex.

In comparing tau immunolabeled tissue preparations with those stained with Thioflavin S, it was evident that profiles labeled with AT8 or PHF-1 were more numerous than the fluorescent profiles seen in Thioflavin S preparations (Fig. 6). However, it was also evident that AT8 appeared to label a larger number of profiles than PHF-1 (Fig. 6; Fig. 7). These findings suggest that while Thioflavin S detects exclusively the filament cores of tau NFTs, AT8 and PHF-1 label the NFTs, as well as portions of the tau fuzzy coat, hyperphosporylated tau, and possibly tau in various states of aggregation.

**Fig. 8:**
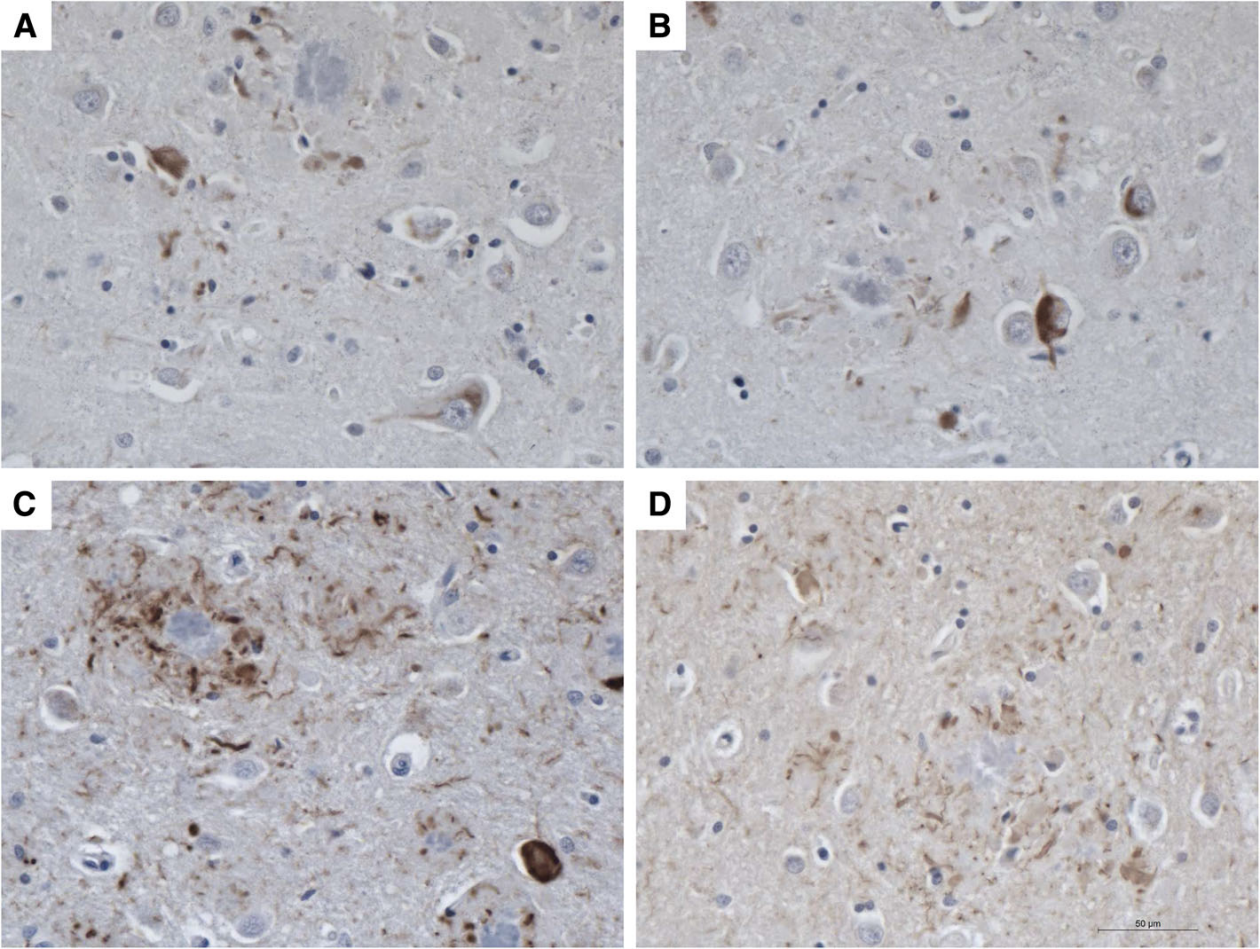
3R and 4R Tau Deposition in the Frontal Lobe of the Moderately to Severely Impaired GSS Patient B. Both 3R (**a**-**b**) and 4R (**c**-**d**) tau were observed in GSS Patient B, although the 4R tau appeared to be more prevalent

In AD, tau inclusions are immunopositive for 3R and 4R tau [8]. Similarly, the tau aggregates in the GSS patient were immunolabeled by monoclonal antibodies to 3R and to 4R tau; however, the 4R antibody appeared to give a stronger labeling than the 3R (Fig. 8).

Immunohistochemistry for Aβ revealed neither diffuse nor cored plaques. Immunohistochemical preparations using antibodies to TDP-43 and α-synuclein did not show intracellular inclusions in neurons or glia.

**Fig. 9:**
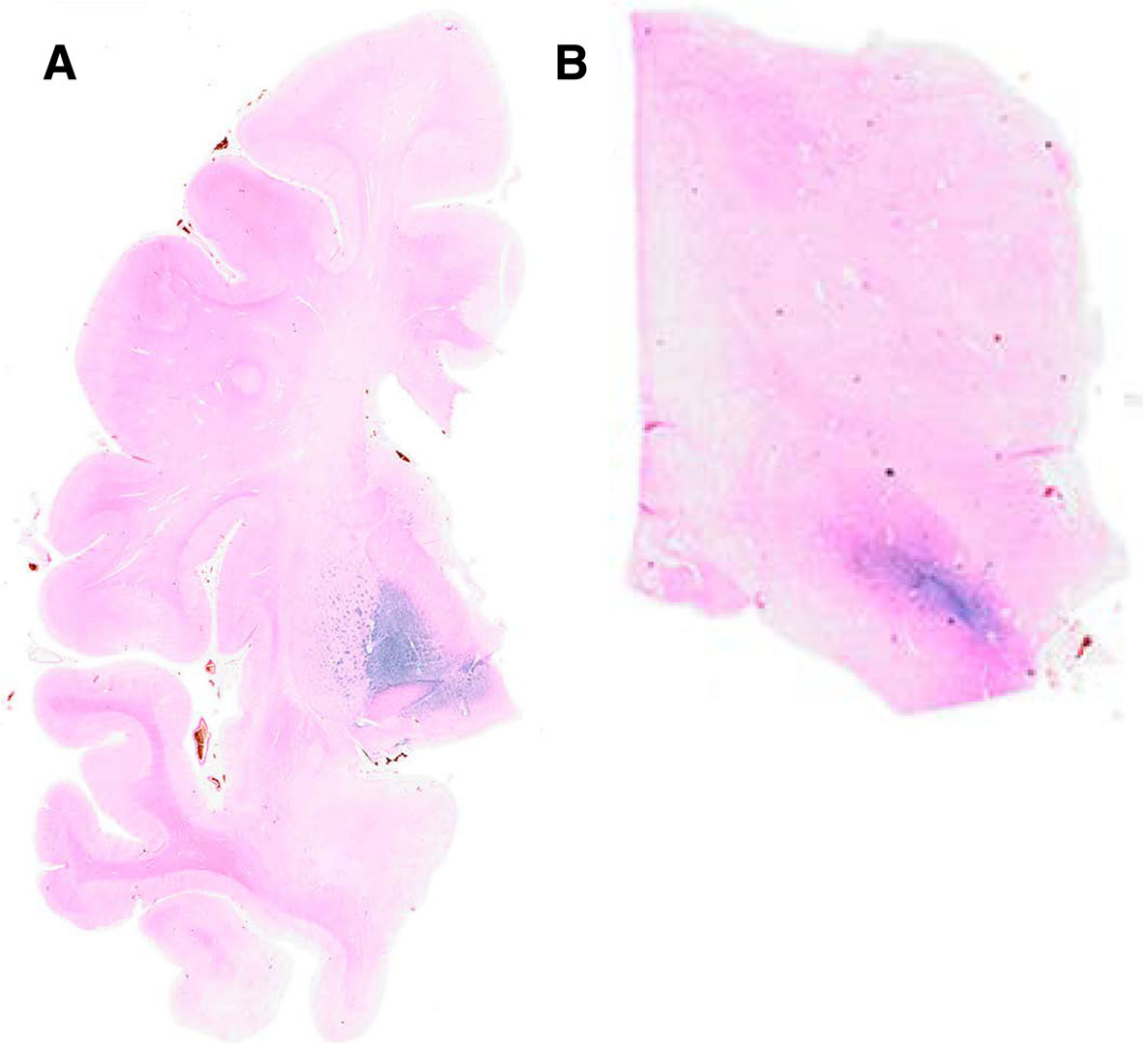
Iron Deposition in the Moderately to Severely Impaired GSS Patient B. Significant iron accumulation is seen in the globus pallidum, as well as in the caudate and putamen (**a**). Iron is also observed in the substantia nigra, while minimal iron binding is observed in the thalamus (**b**)

Similar to what was observed previously in GSS F198S affected individuals [11], ferric iron deposits in the brain of Patient B were most abundant in the globus pallidus and the substantia nigra (Fig. 9a-b), however at the microscopic examination they were detected also in the caudate nucleus and putamen. Iron deposits were not detected in the thalamus (Fig. 9b).

## Discussion

The PET tracer [^18^F]flortaucipir was used to investigate the pattern of tau deposition in two GSS patients who carry the *PRNP* F198S mutation and are members of a pedigree that has been previously studied extensively from the clinical and neuropathological points of view [7, 11, 14, 35].

GSS caused by the *PRNP* F198S mutation is a PrP amyloidosis associated with severe tau deposition in all regions of the cerebrum and brainstem in which misfolded PrP amyloid is observed. On the contrary, tau does not aggregate in the cerebellum, in spite of the heavy PrP burden. DNA changes in the *PRNP gene*, including missense, nonsense, insertion, and deletion mutations, may be associated with a PrP amyloidosis that coexists with severe tau deposition [1, 10, 14, 17, 20, 26, 31]. Patients with different forms of dominantly inherited PrP amyloidosis may present with neuropsychiatric manifestations including depression, personality changes, psychosis, and hallucinations, as well as with frontotemporal dementia-like phenotypes, similar to those previously observed in some *PRNP* F198S mutation carriers [7, 21, 30]. Therefore, it is notable that the two GSS *PRNP* F198S carriers reported in the present study had neurological signs that were preceded by psychiatric symptoms.

This study reveals for the first time that there is a considerable uptake of [^18^F]flortaucipir in various brain regions of both GSS patients with the *PRNP* F198S mutation. Specifically, [^18^F]flortaucipir uptake is evident in the anterior and posterior cingulate gyri, insular cortex, caudate nucleus, nucleus accumbens, putamen, globus pallidus, thalamus, and entorhinal cortex. Furthermore, in comparing the more affected individual with the less affected one, the uptake in the former was greater than that in the latter in the majority of the regions mentioned above. This suggests that a correlation between clinical severity and [^18^F]flortaucipir uptake may exist and that greater [^18^F]flortaucipir uptake may correlate with a higher burden of aggregated tau.

Similar to what occurs in AD, the NFT present in symptomatic *PRNP* F198S mutation carriers are made of 3R and 4R tau; however, the anatomical pattern of [^18^F]flortaucipir PET uptake differs considerably from that seen in early- and late-onset AD [5, 18, 28, 29, 32]. In AD, tau pathology as visualized by [^18^F]flortaucipir PET, appears to be distributed over widespread brain areas in the amnestic, non-amnestic, behavioral, corticobasal, and posterior cortical atrophy variants of the disease [29]. Further, tau PET patterns have been shown to reflect the variability of clinical syndromes and topography of pathologic regions [29]. Many of the anatomical regions found to have high [^18^F]flortaucipir uptake in *PRNP* F198S GSS patients belong to the salience network, as well as to the reward system [27]. Reduction in the function of these networks has been associated with numerous neuropsychiatric disorders, drug dependence, and psychosis. Taken together, our findings and those reported in the literature suggest that in GSS patients carrying the *PRNP* F198S allele the behavioral, psychiatric, and neurological symptoms, as well the dementia that develops in the late stage, result from the interaction of misfolded PrP amyloid and tau. The spreading of tau throughout the entire cerebral cortex and subcortical nuclei contributes to the progression and severity of the psychiatric, neurologic, and cognitive dysfunctions [7, 11, 12, 14].

The neuropathologic results observed in Patient B support the concept that [^18^F]flortaucipir uptake likely reflects the anatomical localization of misfolded tau and NFT that are distributed in the cerebral gray structures. In fact, there is a definite topographic correspondence between [^18^F]flortaucipir uptake and the extent of fluorescent NFT in Thioflavin S preparations of cingulate gyrus, caudate nucleus, putamen, and insular cortex. Immunohistochemical preparations of adjacent serially cut sections of these anatomical regions also revealed a strong immunoreactivity to monoclonal antibodies AT8 and PHF-1 in the cingulate gyrus, caudate nucleus, putamen, and insular cortex. It is also important to note that no Aβ immunoreactivity was present in any CNS area affected by PrP or tau pathology.

A finding that needs to be emphasized and that is relevant to the mechanisms of tau spread and to the correlations between [^18^F]flortaucipir PET images with those obtained by neuropathologic studies is that many CNS gray matter regions demonstrated notable tau immunopositivity, but not an increased signal on PET images. This discrepancy may be due to differences in tau species in these regions, as [^18^F]flortaucipir is known to recognize the NFT, similarly to what Thioflavin S recognizes in histological preparations. Whether [^18^F]flortaucipir may detect states of tau aggregation that precede NFT formation needs further investigation. Thus, tau immunolabeled tissue preparations and those stained with Thioflavin S reveal presence of tau in different states of aggregation. AT8 or PHF-1 reveal the hyperphosphorylated tau burden, which is known to be more widespread than that represented by the fluorescent profiles detected in Thioflavin S preparations. The process of tau becoming hyperphosphorylated is not completely understood; however, this process is known to precede tangle formation. Thus, while Thioflavin S detects only the NFTs made of aggregated tau filament cores, by knowing which tau epitopes are recognized by AT8 or PHF-1, we can conclude that the labeling observed in the specific immunohistochemical preparations recognizes neurons containing not only NFT but also portions of the tau fuzzy coat. It is also possible that the inherent spatial and sensitivity limitations of PET imaging may lead to an inability to detect low levels of tau deposition in vivo. Novel tracers with greater specificity for particular tau filaments are needed [15].

Another region lacking good correspondence between PET imaging and immunohistochemistry is the thalamus, where [^18^F]flortaucipir uptake is relatively strong in both patients, but is weak in the immunohistochemical preparation for tau in GSS Patient B. It should be noted that the thalamus and cerebellum are strongly labeled by immunohistochemistry for PrP; however, no tau immunopositivity is seen in the cerebellum and only a weak immunopositivity is detected in the thalamus. The [^18^F]flortaucipir PET signal in the thalamus may represent “off-target” binding similarly to what has been reported in other studies with [^18^F]flortaucipir tracer showing binding to neuromelanin-containing cells and other targets [22–24]. In view of published studies that showed high iron binding in GSS patients [11] and non-specific binding of [^18^F]flortaucipir to iron [3], we studied neuropathologically Patient B using a method to detect iron deposits, and showed that iron deposits occur mostly in the globus pallidus and the substantia nigra, but not in the thalamus. Therefore, additional studies will be needed to clarify the significance of the uptake of [^18^F]flortaucipir in the thalamus.

Overall, the present study shows for the first time that [^18^F]flortaucipir detects in vivo the severe neurofibrillary pathology that is a significant neuropathologic phenotype in GSS patients carrying the *PRNP* F198S mutation, which potentially has a profound impact on the pathogenesis of psychiatric and neurological symptoms of the disease [11, 12, 14]. Based on evidence obtained in two unpublished presymptomatic mutation carriers, PrP amyloid deposits are detected prior to the occurrence of tau pathology and NFT formation; however, additional in vivo evidence is needed to confirm the PrP-tau relationship and its temporal evolution [11].

In conclusion, it is shown that deposits of tau are detected in vivo by [^18^F]flortaucipir PET in patients who carry the *PRNP* F198S mutation and that tau accumulates with a pattern that is strikingly different from that seen in AD. The patterns of tau anatomical spread seen in GSS *PRNP* F198S and AD may reflect the mechanisms of PrP and Aβ distribution. The results of this study support the view that tau pathology, and not just PrP, contributes significantly to both the psychiatric and the motor symptoms that characterize the phenotype of GSS *PRNP* F198S and that the different clinical phenotypes of GSS *PRNP* F198S and AD correlate with the specific pattern of tau anatomical distribution seen in each disease.

Future studies, comparing in vivo longitudinal PET imaging with the *post-mortem* PrP and tau immunolabeled preparations may allow a precise assessment of the spread of the abnormally conformed proteins over time during the evolution of the *PRNP* F198S GSS disease process.

## Additional files

Additional file 1: **Figure S1.** PrP and Tau in the Thalamus of the Moderately to Severely Impaired GSS Patient B**.** PrP amyloid plaques were also observed in the thalamus of GSS Patient B using Thioflavin S (A). Significant immunolabeling of PrP amyloid (A; 3F4) was also observed. Diffuse hyperphosphorylated tau was observed using AT8, although the burden was considerably less than that seen in the other regions of the brain (C-D). (TIF 3921 kb)

Additional file 2: **Table S1.** Regional [^18^F]Flortaucipir SUVR in *PRNP* F198S GSS Patients Relative to Early-Onset Alzheimer’s Disease Patients and Cognitively Normal Older Adults. (DOCX 24 kb)

## Abbreviations

3R: 3-repeat
4R: 4-repeat
AD: Alzheimer’s disease
ADNI: Alzheimer’s disease neuroimaging initiative
Aβ: Amyloid β
CDR: Clinical Dementia Rating
DWI: Diffusion-weighted imaging
EOAD: Early-onset Alzheimer’s disease
FAS: Functional Assessment Scale
FDDNP: [^18^F]2-(1-(6-[(2-[fluorine-18]fluoroethyl)(methyl)amino]-2-naphthyl)-ethylidene)malononitrile
FDG: [^18^F]Fluorodeoxyglucose
FLAIR: Fluid-attenuated inversion recovery
FWHM: Full-width half maximum
GDS: Geriatric Depression Scale
GSS: Gerstmann-Sträussler-Scheinker
H&E: Hematoxylin and eosin
IADC: Indiana Alzheimer Disease Center
IHC: Immunohistochemistry
LFB-H&E: Luxol fast blue with hematoxylin & eosin
MCI: Mild cognitive impairment
MINT: Multi-lingual Naming Test
MNI: Montreal Neurologic Institute
MoCA: Montreal Cognitive Assessment
MPRAGE: Magnetization-prepared rapid gradient-echo
MRS: Magnetic resonance spectroscopy
NPI-Q: Neuropsychiatric Inventory Questionnaire
PiB: [^11^C]Pittsburgh Compound B
PrP: Prion protein
PTSD: Post-traumatic stress disorder
RAVLT: Rey Auditory Verbal Learning Test
rsfMRI: Resting-state functional MRI
SPECT: Single photon emission computerized tomography
SPM8: Statistical Parametric Mapping 8
SUVR: Standardized uptake value ratio
TMT-A: Trail Making Test A
TMT-B: Trail Making Test B
UDS-3: Uniform Dataset 3
WAIS: Wechsler Adult Intelligence Scale
