# Supplementary figures and images for: Detection of tau in Gerstmann-Sträussler-Scheinker disease (PRNP F198S) by [18F]Flortaucipir PET
Source: Acta Neuropathol Commun. 2018 Oct 29;6:114. doi: 10.1186/s40478-018-0608-z (PMC6205777; doi:10.1186/s40478-018-0608-z)

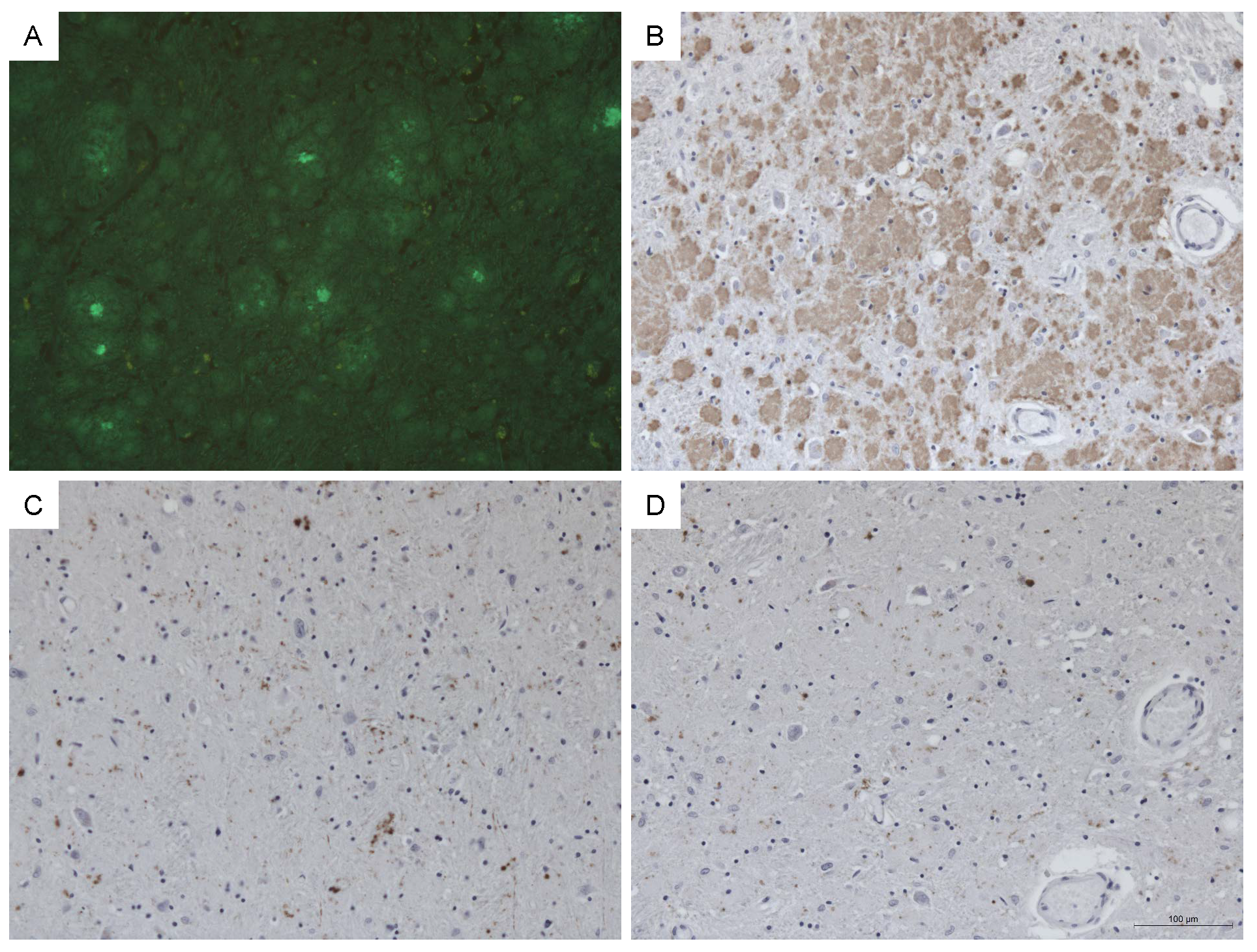

Supplement: Supplementary file 1 — Figure S1. PrP and Tau in the Thalamus of the Moderately to Severely Impaired GSS Patient B. PrP amyloid plaques were also observed in the thalamus of GSS Patient B using Thioflavin S (A). Significant immunolabeling of PrP amyloid (A; 3F4) was also observed. Diffuse hyperphosphorylated tau was observed using AT8, although the burden was considerably less than that seen in the other regions of the brain (C-D). (TIF 3921 kb) [file 40478_2018_608_MOESM1_ESM.tif]
