# Supplementary material for: Detection of tau in Gerstmann-Sträussler-Scheinker disease (PRNP F198S) by [18F]Flortaucipir PET
Source: Acta Neuropathol Commun. 2018 Oct 29;6:114. doi: 10.1186/s40478-018-0608-z (PMC6205777; doi:10.1186/s40478-018-0608-z)
Supplement: Supplementary file 2 — Table S1. Regional [18F]Flortaucipir SUVR in PRNP F198S GSS Patients Relative to Early-Onset Alzheimer’s Disease Patients and Cognitively Normal Older Adults. (DOCX 24 kb) [file 40478_2018_608_MOESM2_ESM.docx]

**Table S1. Regional [^18^F]Flortaucipir SUVR in *PRNP* F198S GSS Patients Relative to Early-Onset Alzheimer’s Disease Patients and Cognitively Normal Older Adults**

|  |  | GSS Patients | |  | Early-Onset  Alzheimer’s | |  | Cognitive Normals | |
| --- | --- | --- | --- | --- | --- | --- | --- | --- | --- |
|  |  | Patient A | Patient B |  | #1 | #2 |  | #1 | #2 |
| Subcortical | Caudate Nucleus | 1.282 | 1.495 |  | 1.291 | 1.251 |  | 1.077 | 1.027 |
|  | Putamen | 1.826 | 2.204 |  | 1.802 | 1.550 |  | 1.372 | 1.328 |
|  | Pallidum | 2.117 | 2.318 |  | 1.893 | 1.595 |  | 1.475 | 1.439 |
|  | Hippocampus | 1.240 | 1.228 |  | 1.935 | 1.786 |  | 1.087 | 1.212 |
|  | Amygdala | 1.267 | 1.261 |  | 2.150 | 2.190 |  | 1.006 | 1.113 |
|  | Thalamus | 1.475 | 1.546 |  | 1.293 | 1.329 |  | 1.218 | 1.240 |
|  | Nucleus Accumbens | 1.616 | 1.582 |  | 1.620 | 1.500 |  | 1.135 | 1.159 |
|  |  |  |  |  |  |  |  |  |  |
| Frontal Lobe | Lateral Orbitofrontal Gyrus | 1.275 | 1.271 |  | 1.687 | 1.608 |  | 1.037 | 1.168 |
|  | Medial Orbitofrontal Gyrus | 1.176 | 1.175 |  | 1.484 | 1.474 |  | 0.947 | 1.048 |
|  | Pars Orbitalis | 1.257 | 1.201 |  | 1.535 | 1.476 |  | 1.052 | 1.126 |
|  | Pars Opercularis | 1.194 | 1.237 |  | 1.869 | 1.65 |  | 1.035 | 1.017 |
|  | Pars Triangularis | 1.192 | 1.167 |  | 1.602 | 1.489 |  | 1.005 | 1.043 |
|  | Middle Frontal Gyrus | 1.140 | 1.105 |  | 1.947 | 1.495 |  | 0.949 | 0.918 |
|  | Frontal Pole | 1.119 | 1.107 |  | 1.303 | 1.095 |  | 0.846 | 0.876 |
|  | Superior Frontal Gyrus | 1.116 | 1.196 |  | 1.781 | 1.256 |  | 0.890 | 0.893 |
|  | Mean Frontal Lobe | 1.183 | 1.182 |  | 1.684 | 1.449 |  | 0.970 | 1.001 |
|  |  |  |  |  |  |  |  |  |  |
| Cingulate | Cingulate Gyrus, Anterior | 1.198 | 1.392 |  | 1.356 | 1.430 |  | 0.957 | 1.000 |
|  | Cingulate Gyrus, Isthmus | 1.143 | 1.076 |  | 2.123 | 2.299 |  | 0.968 | 1.027 |
|  | Cingulate Gyrus, Posterior | 1.273 | 1.414 |  | 2.082 | 2.173 |  | 0.991 | 1.025 |
|  | Mean Cingulate | 1.205 | 1.294 |  | 1.729 | 1.883 |  | 0.972 | 1.013 |
|  |  |  |  |  |  |  |  |  |  |
| Parietal Lobe | Inferior Parietal Lobule | 1.191 | 1.030 |  | 2.616 | 2.628 |  | 1.018 | 1.059 |
|  | Superior Parietal Lobule | 1.273 | 1.043 |  | 1.807 | 2.192 |  | 0.944 | 0.951 |
|  | Supramarginal Gyrus | 1.185 | 0.967 |  | 2.036 | 2.163 |  | 0.991 | 1.006 |
|  | Precuneus | 1.271 | 1.122 |  | 2.085 | 2.980 |  | 1.019 | 1.029 |
|  | Mean Parietal Lobe | 1.230 | 1.040 |  | 2.136 | 2.490 |  | 0.993 | 1.011 |
|  |  |  |  |  |  |  |  |  |  |
| Temporal Lobe | Entorhinal Cortex | 1.250 | 1.361 |  | 1.887 | 1.965 |  | 0.931 | 1.115 |
|  | Fusiform Gyrus | 1.270 | 1.258 |  | 2.316 | 2.051 |  | 1.033 | 1.136 |
|  | Parahippocampal Gyrus | 1.241 | 1.252 |  | 1.974 | 1.675 |  | 0.926 | 1.100 |
|  | Temporal Pole | 1.253 | 1.287 |  | 1.719 | 1.544 |  | 0.939 | 1.202 |
|  | Banks of the Superior  Temporal Sulcus | 1.364 | 1.120 |  | 2.682 | 2.872 |  | 1.113 | 1.104 |
|  | Inferior Temporal Gyrus | 1.249 | 1.229 |  | 2.821 | 2.128 |  | 1.005 | 1.162 |
|  | Middle Temporal Gyrus | 1.260 | 1.161 |  | 2.632 | 2.258 |  | 1.052 | 1.119 |
|  | Superior Temporal Gyrus | 1.205 | 1.119 |  | 1.931 | 1.861 |  | 0.975 | 1.019 |
|  | Transverse Temporal Gyrus | 1.124 | 1.105 |  | 1.386 | 1.611 |  | 1.029 | 1.031 |
|  | Mean Temporal Lobe | 1.246 | 1.210 |  | 2.150 | 1.996 |  | 1.001 | 1.110 |
|  |  |  |  |  |  |  |  |  |  |
| Occipital Lobe | Lateral Occipital Gyrus | 1.164 | 1.090 |  | 1.909 | 1.978 |  | 0.995 | 1.021 |
|  | Cuneus | 1.142 | 1.066 |  | 1.553 | 1.965 |  | 1.036 | 1.032 |
|  | Pericalcarine Gyrus | 1.104 | 1.030 |  | 1.305 | 1.588 |  | 1.029 | 1.064 |
|  | Lingual Gyrus | 1.067 | 1.103 |  | 1.528 | 1.839 |  | 0.928 | 1.030 |
|  | Mean Occipital Lobe | 1.119 | 1.072 |  | 1.589 | 1.843 |  | 0.997 | 1.039 |
|  |  |  |  |  |  |  |  |  |  |
| Sensory-Motor Cortex | Precentral Gyrus | 1.127 | 1.201 |  | 1.376 | 1.282 |  | 0.939 | 0.899 |
|  | Postcentral Gyrus | 1.133 | 1.013 |  | 1.311 | 1.318 |  | 0.910 | 1.025 |
|  | Paracentral Lobule | 1.241 | 1.289 |  | 1.262 | 1.606 |  | 0.933 | 0.953 |
|  | Mean Sensory-Motor Cortex | 1.167 | 1.168 |  | 1.316 | 1.402 |  | 0.927 | 0.908 |
|  |  |  |  |  |  |  |  |  |  |
|  | Insular Cortex | 1.227 | 1.332 |  | 1.599 | 1.571 |  | 1.016 | 1.077 |
|  |  |  |  |  |  |  |  |  |  |
| Cerebellum | Cerebellar Cortex* | 228.190 | 143.222 |  | 301.341 | 164.894 |  | 337.190 | 174.728 |
|  | Cerebellar Crus* | 214.946 | 136.426 |  | 276.019 | 150.042 |  | 363.656 | 168.914 |
|  | Whole Cerebellum* | 229.863 | 142.852 |  | 303.519 | 165.644 |  | 339.337 | 176.004 |

* These values are not normalized to the cerebellar crus and represent SUV values from the smoothed, normalized mean 80-100 images normalized to injected dose.

GSS = Gerstmann-Sträussler-Scheinker disease
